# Combined tenofovir, lamivudine, and dolutegravir treatment increases leptin and FABP4 expression in human subcutaneous adipose tissue

**DOI:** 10.31744/einstein_journal/2026AO1770

**Published:** 2026-02-02

**Authors:** Luiza Wolf Zanardo, Larissa dos Santos Izabel, Letícia Miho Hamada, Marcela Botelho de Carvalho, Pedro Afonso Grandino Oliveira e Silva, Henrique Dametto Giroud Joaquim, Sergio Santoro, William Tadeu Festuccia, Juliana Magdalon

**Affiliations:** 1 Hospital Israelita Albert Einstein Faculdade Israelita de Ciências da Saúde Albert Einstein São Paulo SP Brazil Faculdade Israelita de Ciências da Saúde Albert Einstein, Hospital Israelita Albert Einstein, São Paulo, SP, Brazil.; 2 Hospital Israelita Albert Einstein São Paulo SP Brazil Hospital Israelita Albert Einstein, São Paulo, SP, Brazil.; 3 Universidade de São Paulo Instituto de Ciências Biomédicas São Paulo SP Brazil Instituto de Ciências Biomédicas, Universidade de São Paulo, São Paulo, SP, Brazil.

**Keywords:** Antiretroviral therapy, highly active, HIV, Stromal vascular fraction, Adipose tissue

## Abstract

**Introduction:**

Several antiretroviral regimens reportedly affect adipose tissue function. While earlier antiretroviral combinations were associated with lipodystrophy, more recent regimens, particularly those containing dolutegravir, have been linked to fat gain. However, the direct impact of contemporary antiretroviral regimens on human adipose tissue remains unclear.

**Objective:**

Therefore, this study aimed to elucidate the effects of the combination of TDF, 3TC, and DTG on human adipose tissue function *in vitro*. For comparison, we also analyzed an alternative regimen consisting of AZT, 3TC, and DTG, given the well-documented association of AZT with lipodystrophy.

**Methods:**

Cells from the stromal vascular fraction of both adipose depots were isolated and treated with each antiretroviral combination for 72 hours. Additionally, adipose tissue explants were treated with each regimen for one week *in vitro*.

**Results:**

Both treatments significantly reduced the number of viable stromal vascular fraction cells, as determined by the MTT assay. Moreover, combined TDF, 3TC, and DTG treatment increased FABP4 and leptin expression only in subcutaneous adipose tissue. No significant changes were observed in inflammatory cytokine expression, adiponectin expression and secretion, or lipolysis in either adipose depot treated with the antiretroviral combinations.

**Conclusion:**

These findings suggest that the tested antiretroviral combinations may reduce stromal vascular fraction cell number and that the current first-line antiretroviral combination increases FABP4 and leptin expression in subcutaneous adipose tissue, indicating the potential impact of continued antiretroviral use on systemic metabolism in people living with HIV.

## In Brief

Zanardo et al. showed *in vitro* that the first-line HIV regimen (tenofovir disoproxil fumarate, lamivudine, dolutegravir) decreases the number of viable stromal vascular fraction cells in subcutaneous and visceral adipose tissues and increases leptin and FABP4 expression in subcutaneous adipose tissue, indicating potential impacts on adipose tissue function.

## Highlights

■ TDF + 3TC + DTG decreases the number of viable stromal vascular cells in subcutaneous adipose tissue.
■ TDF + 3TC + DTG decreases the number of viable stromal vascular cells in visceral adipose tissue.
■ TDF + 3TC + DTG increases leptin and FABP4 expression in subcutaneous adipose tissue.

## Graphical abstract

**Figure:**
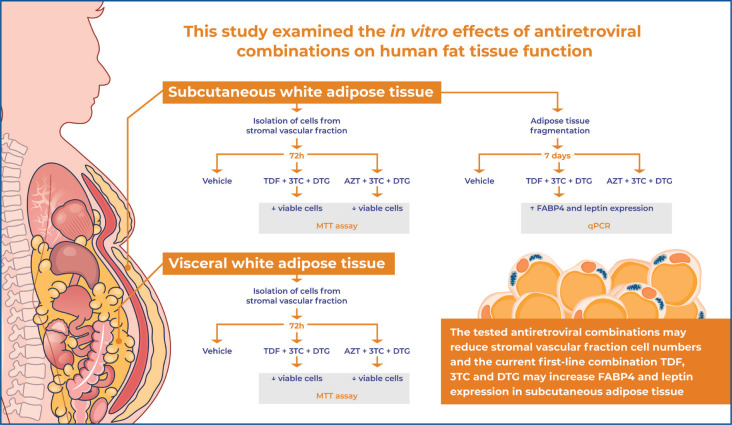

## INTRODUCTION

The human immunodeficiency virus (HIV) was discovered in the 1980s in the context of successive diagnoses of opportunistic infections in individuals previously considered healthy. It was later determined that these individuals had developed acquired immunodeficiency due to the HIV infection.^(1,2)^ Although the exact origin of the virus remains unknown, it is believed to have originated from non-human primates and to have begun transmission to humans in the early 1900s.^(3)^

Four decades after its discovery and the onset of the HIV pandemic, the incidence of new infections reached its lowest point in 2023 since the late 1980s. Globally, 39% fewer people were infected with HIV compared to the figure in 2010, primarily due to the swift response of the scientific community in advancing antiretroviral therapies and treatment distribution policies.^(4)^ Antiretroviral treatment plays a crucial role in reducing HIV incidence, as individuals with an undetectable viral load do not transmit the virus.

Zidovudine (AZT), a nucleoside reverse transcriptase inhibitor (NRTI), was the first antiretroviral drug to be studied and approved for the treatment of HIV in 1987, with its significance further reinforced in 1994 when studies demonstrated its effectiveness in preventing vertical transmission.^(1)^ In 1995-96, new classes of antiretroviral drugs were introduced, enabling multidrug regimens that proved to be highly effective.^(5)^ However, earlier antiretrovirals, including AZT, are associated with significant side effects, such as lipodystrophy.^(6)^ This condition is characterized by the loss and redistribution of body fat, including lipoatrophy in the subcutaneous adipose tissue of the upper and lower limbs, buttocks, and face, accompanied by lipohypertrophy in the neck ("buffalo hump"), trunk, and intra-abdominal region, leading to visceral adipose tissue accumulation.^(7)^ Importantly, in HIV-infected patients undergoing treatment, reduced muscle mass and increased central adiposity appear to be significant risk factors for mortality.^(8)^

Following the identification of these adverse effects, newer antiretroviral drugs with reduced toxicity to adipocytes were developed, and the widespread use of antiretroviral therapy, resulting in effective viral suppression, has significantly increased the life expectancy of people living with HIV.^(9)^ In this evolving therapeutic context, the primary causes of mortality are increasingly linked to aging-related conditions, such as cancer and cardiovascular diseases.^(10–12)^ Notably, many current antiretroviral drugs, particularly integrase strand transfer inhibitors (INSTIs), are associated with weight gain and increased accumulation of visceral adipose tissue.^(13,14)^

White adipose tissue (WAT), which is categorized based on anatomical location into visceral and subcutaneous depots, performs multiple functions and is associated with various metabolic risks contingent upon its absolute quantity and specific distribution.^(15)^ The primary function of WAT is the long-term storage of energy in the form of triacylglycerol within adipocytes. Beyond energy storage, WAT is increasingly recognized for its endocrine functions, as it synthesizes and secretes a range of bioactive molecules known as adipokines, such as adiponectin and leptin. These adipokines exert both local and systemic effects, regulating metabolic processes, such as insulin sensitivity, and modulating inflammatory responses.^(16)^ Visceral adipose tissue, in particular, exhibits elevated production of pro-inflammatory adipokines, such as tumor necrosis factor alpha (TNF-α) and interleukin (IL)-6, and contains a higher density of immune cells than subcutaneous adipose tissue.^(17,18)^ Consequently, increased visceral adipose tissue is strongly correlated with the onset of metabolic disorders.^(19)^

Regarding current antiretroviral therapy regimens for adults, the World Health Organization recommends a combination of two nucleoside/nucleotide reverse transcriptase inhibitors (NRTI/NtRTI), with a preference for tenofovir disoproxil fumarate (TDF) and lamivudine (3TC), alongside a third agent from a different class, such as a non-NRTI (NNRTI), protease inhibitor (PI), or INSTIs, with dolutegravir (DTG) as the preferred choice. Although several clinical studies have shown alterations in the WAT of patients treated with different antiretroviral combinations, no *in vitro* study has specifically investigated the effects of this first-line regimen on WAT. Consequently, its potential direct impact on this tissue remains unexplored.

## OBJECTIVE

Therefore, this study aimed to elucidate the effects of the combination of TDF, 3TC, and DTG on human adipose tissue function *in vitro*. For comparison, we also analyzed an alternative regimen consisting of AZT, 3TC, and DTG, given the well-documented association of AZT with lipodystrophy.

## METHODS

### Ethical considerations

This study was approved by the Research Ethics Committee of the *Hospital Israelita Albert Einstein* (CAAE: 15007719.4.0000.0071; #6.602.854). Samples were collected from 12 voluntary participants over the age of 18 years who provided informed consent.

### Sample collection and experimental groups

Approximately 1g of subcutaneous and visceral (omentum) abdominal WAT was collected from patients undergoing bariatric surgery or other elective abdominal surgeries. After collection, the samples were transported to the laboratory in sealed containers, where part of the tissue fragments was used for isolating cells from the stromal vascular fraction (SVF), while another part was used for adipose tissue explant culture. Three experimental groups were established for both cell and explant culture: 1) control (vehicle); 2) treatment with TDF + 3TC + DTG; and 3) treatment with AZT + 3TC + DTG. The drug concentrations were selected to reflect peak plasma concentrations (Cmax) observed in patients undergoing treatment: 0.3*μ*g/mL TDF (0.58*μ*M),^(20)^ 2*μ*g/mL 3TC (8.72*μ*M),^(21,22)^ 3.5 *μ*g/mL DTG (8.35*μ*M),^(23)^ and 1.2 *μ*g/mL AZT (4.49 *μ*M).^(24–26)^

### Isolation of cells from the stromal vascular fraction

Human WAT-derived SVF cells were isolated by the addition of collagenase.^(27)^ Approximately 1 g of minced tissue was washed with 10 mL of phosphate-buffered saline (PBS) and then digested with 1 mg/mL of type I collagenase for 30-60 minutes, while continuously shaking at 37°C. Subsequently, the material was filtered through a 100 *μ*m cell strainer and centrifuged at 800g for 10 minutes at 22°C. The supernatant was discarded, and the cells were washed with 15 mL of red blood cell lysis buffer. Subsequently, a second centrifugation was performed at 800g for 10 minutes at 22°C, and the supernatant was discarded. The cells were then resuspended in Dulbecco's Modified Eagle Medium/Nutrient Mixture F-12 (DMEM/F12) culture medium supplemented with 10% fetal bovine serum (FBS) and 1% penicillin/streptomycin (P/S) and cultured in Petri dishes at 37°C with 5% CO_2_. Only one lot of FBS was used. The medium was changed after 2 days, and subsequently every 3 to 4 days until the cells reached approximately 80% confluence, at which point they were detached from the plate using trypsin and subcultured on new plates (passage 1). The cells were maintained under the same conditions until a maximum of passage 4 was reached.

### Quantification of viable cells

The MTT assay was used to analyze the number of viable cells. Briefly, an equal number of cells (10,000 cells per well) were added to 96-well plates in the presence of vehicle, TDF + 3TC + DTG, or AZT + 3TC + DTG (in triplicate), resulting in a final volume of 90*μ*L in each well. After 69 hours, 10*μ*L of 5mg/mL MTT (Sigma-Aldrich) was added, and the plates were incubated for an additional 3 hours at 37°C, completing a total treatment time of 72 hours. Subsequently, the supernatants were discarded, and 50*μ*L of 0.1 N HCl in anhydrous isopropanol was added. After dissolution of the formazan crystals, absorbance was measured at 570 nm and 690 nm and plotted as the difference after subtracting the blank (well without cells) absorbance value.

### Adipose tissue explant culture

Human WAT explants were cultured in accordance with the protocol established by Carswell et al.^(28)^ Fragments of adipose tissue were cultured in plates for 7 days. Initially, the tissues were minced into pieces weighing 5-10mg (1-2mm³) and subsequently placed in 100*μ*m cell stainers to wash with PBS for removing debris and lipids released by the cells. The explants were then transferred to 6-well culture plates, using 50-100mg of tissue per well. A total of 2 mL of culture medium was added per well, consisting of DMEM/F12 supplemented with 10% FBS, 1% P/S, 5*μ*g/mL insulin, and 1*μ*M dexamethasone, in the presence of vehicle, TDF + 3TC + DTG, or AZT + 3TC + DTG. The culture medium, along with the treatments, was changed once during the 7-day period, three days after the initiation of the culture.

### RNA extraction, reverse transcription, and gene expression

Total RNA was extracted from the tissues after treatment for 7 days with vehicle, TDF + 3TC + DTG, or AZT + 3TC + DTG using TRIzol reagent (ThermoFisher Scientific), following the manufacturer's instructions. Reverse transcription was performed using 1*μ*g of total RNA with the High-Capacity cDNA Reverse Transcription Kit (ThermoFisher Scientific) according to the manufacturer's recommendations. Quantitative PCR (qPCR) was conducted using the SYBR Green PCR master mix (ThermoFisher Scientific), and the expression levels of the following genes were evaluated: *TNF*-α, *IL*-6, *IL-10*, fatty-acid binding protein 4 (*FABP4*), leptin and adiponectin. The primers used are detailed in table 1. The results for each sample of target genes were normalized to the mean expression of two reference genes (β*2M* and *HPRT1*) and expressed relative to that of the control group.

**Table 1:** Primers used for real-time polymerase chain reaction

Gene	Forward Primer (5’ – 3’)	Reverse Primer (5’ – 3’)
TNF-α	CCCATGTTGTAGCAAACCCTC	TATCTCTCAGCTCCACGCCA
IL-6	TTCTCCACAAGCGCCTTC	TGAAGAGGTGAGTGGCTGTC
IL-10	TGCCTTTAATAAGCTCCAAGAGAAA	AGAGTCGCCACCCTGATGTC
FABP4	GGAAAGTCAAGAGCACCATAACC	AACTCTCGTGGAAGTGACGC
LEP	GTGCGGATTCTTGTGGCTTT	GAGACTGACTGCGTGTGTGAA
ADIPOQ	AAGGAGATCCAGGTCTTATTGGTCC	CACACTGAATGCTGAGCGGTA
β2M	AGATGAGTATGCCTGCCGTG	TTCAAACCTCCATGATGCTGC
HPRT1	CTTCCTCCTCCTGAGCAGTC	TCATCACTAATCACGACGCCA

ADIPOQ: Adiponectin. β2M: Beta-2 microglobulin. FABP4: Fatty-acid binding protein 4. HPRT1: Hypoxanthine guanine phosphoribosyltransferase. IL-6: Interleukin 6. IL-10: Interleukin 10. LEP: Leptin. TNF-α: Tumor necrosis factor alpha.

### Adiponectin secretion and lipolysis

Supernatant was collected from adipose tissue explant culture incubated with vehicle, TDF + 3TC + DTG, or AZT + 3TC + DTG for the last four days of the 7-day period of treatment. Adiponectin secretion was evaluated by enzyme-linked immunosorbent assay (Adiponectin ELISA kit, Millipore) according to the manufacturer's instructions. Lipolysis was analyzed by measuring free glycerol (Free Glycerol Kit, Sigma-Aldrich). The results were normalized to the total protein concentrations—analyzed by Pierce BCA Protein Assay (Thermo-Scientific) following supplier recommendation—present in the supernatants.

### Statistical analysis

Data are expressed as mean ± standard error. Data normality was assessed using the Shapiro-Wilk test. Parametric data were analyzed using one-way repeated measures ANOVA followed by Tukey's post hoc test, while non-parametric data were analyzed using the Friedman test followed by Dunn's post hoc test. Statistical significance was set at p<0.05. GraphPad Prism 10.3.1^®^ (GraphPad Software Inc.) was used for statistical analysis.

## RESULTS

**Figure 1:**
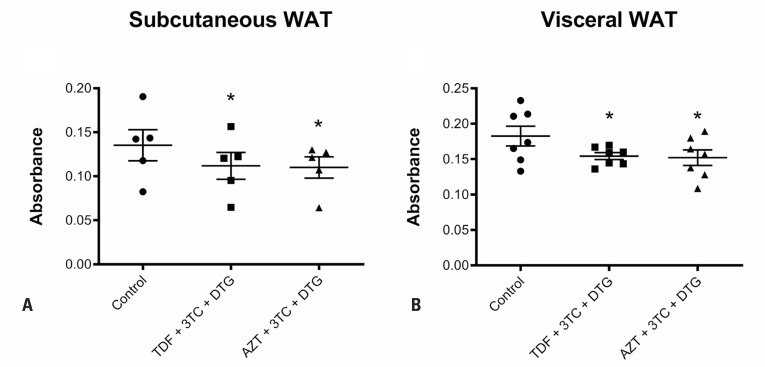
Analysis of viable cells isolated from the stromal vascular fraction (SVF) of subcutaneous (A) and visceral (B) white adipose tissue (WAT) using MTT assay after treatment for 72 hours with vehicle; tenofovir disoproxil fumarate (TDF) + lamivudine (3TC) + dolutegravir (DTG); or zidovudine (AZT) + 3TC + DTG

In subcutaneous WAT, both combinations—TDF + 3TC + DTG and AZT + 3TC + DTG—induced a significantly lower number of viable SVF cells than the control by 17% (p=0.0185) and 19% (p=0.0126), respectively, as assessed by MTT assay (Figure 1A). Similarly, both treatments significantly reduced the number of viable visceral WAT cells by 15% (p=0.0181) and 17% (p=0.0114) (Figure 1B), respectively, compared to that by the control.

*p<0.05 compared to the control.

Results are presented as mean ± SEM. One-way repeated measures ANOVA followed by Tukey's post hoc test was employed for statistical analysis.

**Figure 2:**
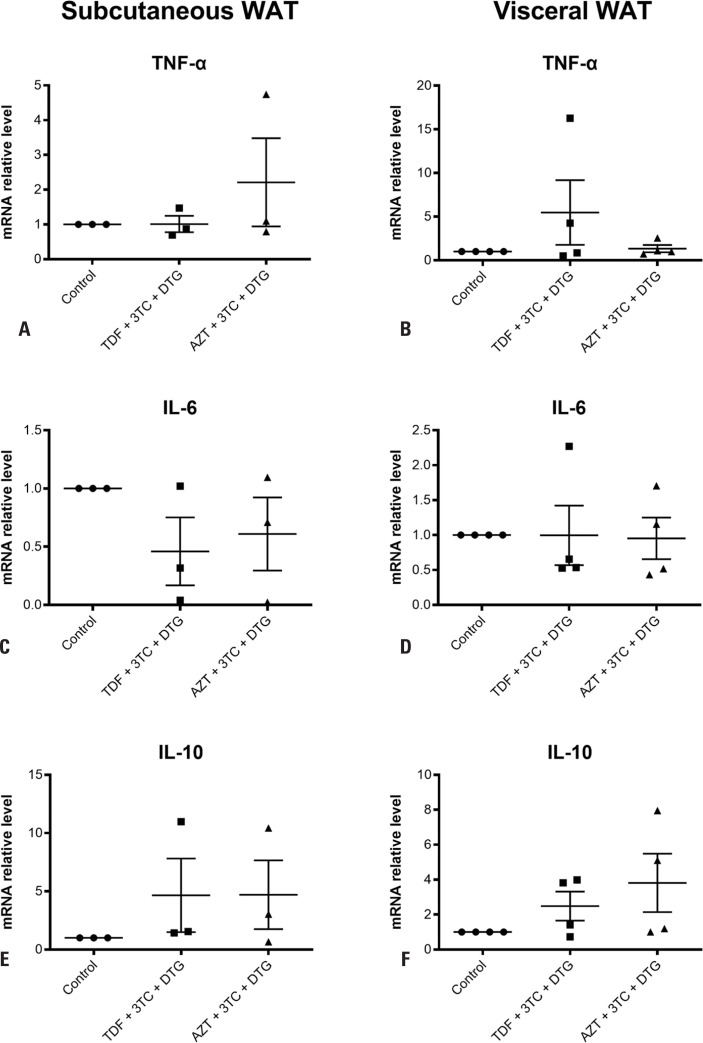
Analysis of relative mRNA levels of *TNF*-α (A, B), *IL-6* (C, D), and *IL-10* (E, F) in subcutaneous and visceral white adipose tissue (WAT) using qPCR after treatment for 7 days with vehicle; tenofovir disoproxil fumarate (TDF) + lamivudine (3TC) + dolutegravir (DTG); or zidovudine (AZT) + 3TC + DTG

**Figure 3:**
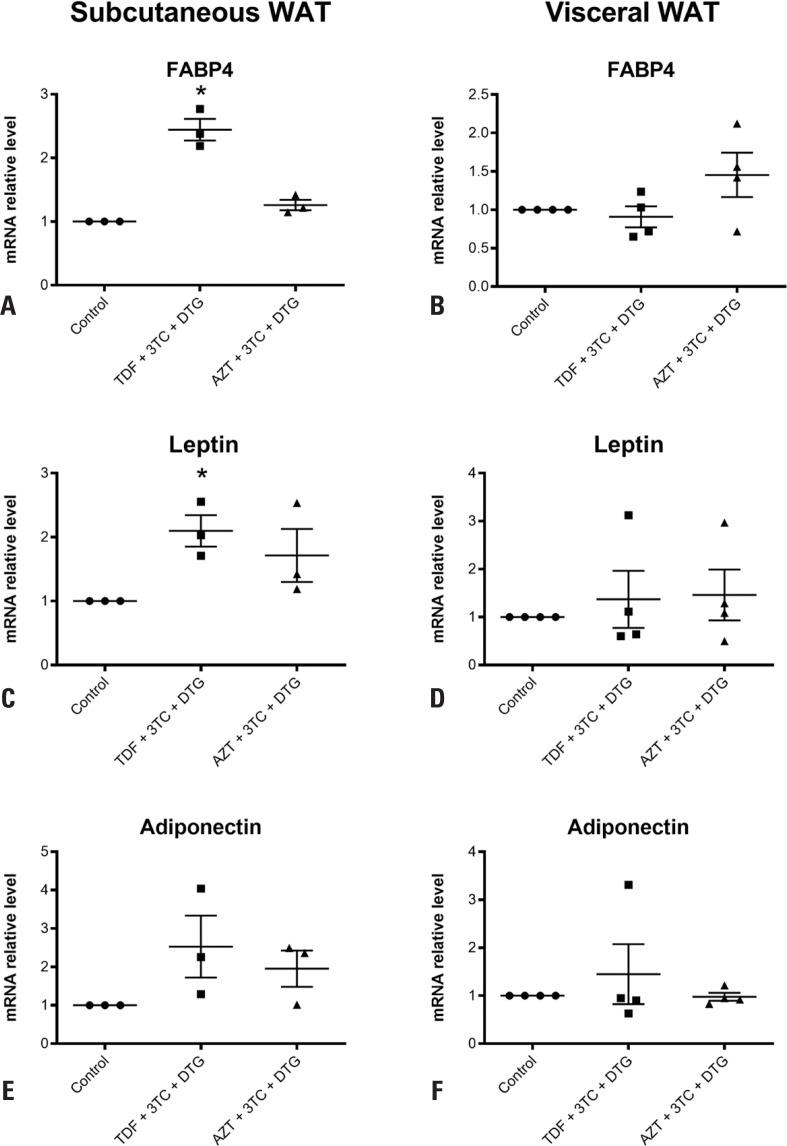
Analysis of relative mRNA levels of *FABP4* (A, B), leptin (C, D), and adiponectin (E, F) in subcutaneous and visceral white adipose tissue (WAT) using qPCR after treatment for 7 days with vehicle; tenofovir disoproxil fumarate (TDF) + lamivudine (3TC) + dolutegravir (DTG); or zidovudine (AZT) + 3TC + DTG

We next examined the effect of the treatments on gene expression. The gene expression of the inflammatory cytokines TNF-α (Figure 2A, 2B), IL-6 (Figure 2C, 2D), and IL-10 (Figure 2E, 2F) was not altered by TDF + 3TC + DTG or AZT + 3TC + DTG in both subcutaneous and visceral WAT. However, TDF + 3TC + DTG increased FABP4 and leptin expression in subcutaneous WAT but not visceral WAT by 2.4-fold (p=0.0286) and 2.1-fold (p=0.0286), respectively, compared to the control (Figure 3A, 3B, 3C, 3D). FABP4 and leptin expression was also not altered by AZT + 3TC + DTG in both subcutaneous and visceral WAT (Figure 3A, 3B, 3C, 3D).

Results are presented as mean ± SEM. Friedman test followed by Dunn's post hoc test was employed for statistical analysis.

*p<0.05 compared to the control.

Results are presented as mean ± SEM. Friedman test followed by Dunn's post hoc test was employed for statistical analysis.

**Figure 4:**
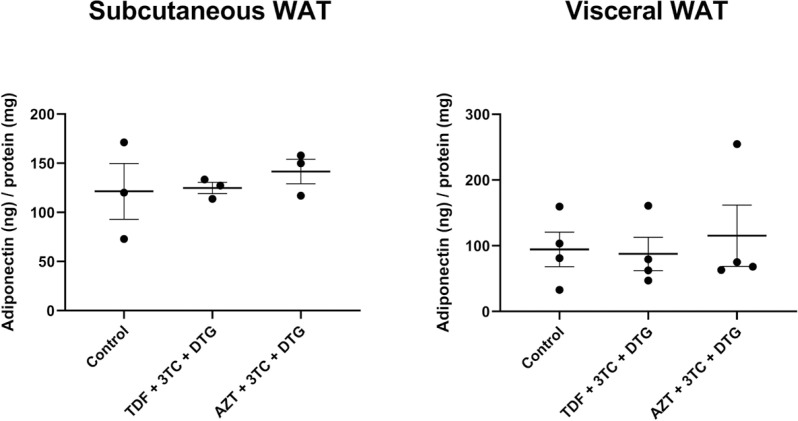
Analysis of adiponectin secretion from subcutaneous (A) and visceral (B) white adipose tissue (WAT) using ELISA after treatment for 7 days with vehicle; tenofovir disoproxil fumarate (TDF) + lamivudine (3TC) + dolutegravir (DTG); or zidovudine (AZT) + 3TC + DTG

**Figure 5:**
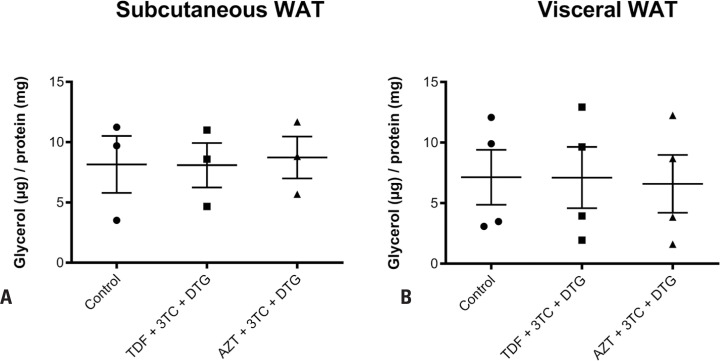
Analysis of lipolysis in subcutaneous (A) and visceral (B) white adipose tissue (WAT) via glycerol quantitation in the supernatant after treatment for 7 days with vehicle; tenofovir disoproxil fumarate (TDF) + lamivudine (3TC) + dolutegravir (DTG); or zidovudine (AZT) + 3TC + DTG

Further, the results indicated that adiponectin expression was not changed by any of the treatments compared to that by the control (Figure 3E, 3F). Similarly, treatment with TDF + 3TC + DTG or AZT + 3TC + DTG induced no alteration in adiponectin secretion compared to that by the control in both subcutaneous and visceral WAT, as assessed by ELISA (Figure 4A, 4B). Moreover, treatment with TDF + 3TC + DTG or AZT + 3TC + DTG induced no alteration in lipolysis compared to that by the control in both subcutaneous and visceral adipose tissue, as measured by glycerol content in the supernatant (Figure 5A, 5B).

Results are presented as mean ± SEM. Friedman test followed by Dunn's post hoc test was employed for statistical analysis.

Results are presented as mean ± SEM. Friedman test followed by Dunn's post hoc test was employed for statistical analysis.

## DISCUSSION

Antiretroviral therapy has significantly reduced HIV-related mortality. Over the years, considerable progress has been made in developing drugs capable of suppressing viral replication while minimizing adverse effects. Despite these advancements, many antiretroviral drugs reportedly impact adipose tissue, inducing conditions, such as lipoatrophy, lipodystrophy, or fat accumulation, given their direct effects on the WAT, including adipocytes and SVF cells.^(6)^ However, limited information is available regarding the direct effects of the current first-line antiretroviral combination, TDF + 3TC + DTG, on adipose tissue. To address this gap, the present study evaluated the *in vitro* effects of this combination on subcutaneous and visceral WAT. For comparison, we also investigated an alternative combination, consisting of AZT + 3TC + DTG, given the well-documented association of AZT with lipoatrophy or lipodystrophy. Our findings indicated that both antiretroviral combinations reduced the number of viable SVF cells from subcutaneous and visceral WAT. Moreover, TDF + 3TC + DTG increased both FABP4 and leptin expression only in subcutaneous WAT. However, neither combination elicited changes in inflammatory cytokine gene expression, adiponectin expression and secretion, or lipolysis after one week of treatment, suggesting minimal effects on adipose tissue under short-term exposure conditions.

The SVF of adipose tissue comprises mesenchymal stem cells and preadipocytes, which may differentiate into mature adipocytes upon exposure to pro-adipogenic stimuli.^(15)^ No alteration in cell number was observed in 3T3-F442A preadipocyte cells induced to differentiate into adipocytes for 7 days and treated with TDF or 3TC alone; however, it was found that AZT alone or combined with 3TC reduced the number of viable cells, as measured by MTT assay, and that AZT alone induced apoptosis in these cells.^(29)^ Similarly, the proliferation of the human preadipocyte cell line Simpson-Golabi-Behmel Syndrome (SGBS) remained unchanged when treated with TDF alone, but a mild reduction in cell number was observed when TDF was combined with DTG,^(30)^ supporting our findings. These discrepancies might be attributed not only to the antiretroviral combinations but also differences in the study models, as TDF alone has also been reported to reduce the number of SVF cells from human adipose tissue, as measured by MTT assay, which is consistent with our study.^(31)^ Notably, the MTT assay measures cellular metabolic activity and serves as an indirect indicator of the number of viable cells, which can reflect an alteration in cell proliferation or cell death. Importantly, this reduction in cell number was observed after only one week of antiretroviral exposure. Although the long-term effects remain unknown, prolonged treatment could potentially exacerbate these changes, with possible implications for adipose tissue function and systemic metabolism.

The observed reduction in SVF cell number following treatment with both antiretroviral combinations could suggest a potential inhibition of adipogenesis. However, contrary to expectations, the combination of TDF + 3TC + DTG increased the expression of *FABP4*—a key gene expressed by mature adipocytes—in subcutaneous WAT, suggesting increased adipogenesis. This is in line with the observation that patients on DTG tend to gain weight, although they mainly accumulate visceral WAT. Similar to our findings, when DTG alone was added to SVF cells from subcutaneous WAT, adipogenesis was stimulated, as shown by greater lipid accumulation and higher expression of pro-adipogenic markers, such as FABP4.^(32)^ However, when DTG was added to SGBS preadipocytes, alone or in combination with TDF, it reduced the expression of key adipocyte genes, including lipoprotein lipase and *PPAR*γ, suggesting adipogenesis inhibition.^(30,33)^ Such discrepancies may result from cell-specific effects, as DTG has also been shown to inhibit beige adipocyte differentiation.^(34,35)^ Other antiretrovirals have been shown to affect adipocyte differentiation. For example, while TDF or 3TC alone has no reported impact on adipogenesis,^(29,31)^ the combination of TDF with the NRTI emtricitabine and either the NNRTI efavirenz or the PI atazanavir has been shown to inhibit the differentiation of SVF cells into mature adipocytes.^(31)^ Interestingly, when evaluating 3T3-F442A preadipocyte differentiation into adipocytes by measuring the expression of malic enzyme and fatty acid synthase after 11 days, AZT alone exerted an inhibitory effect, but combining it with 3TC increased their expression, suggesting enhanced adipogenesis.^(36)^ In contrast, in our study, no alteration was observed in FABP4 expression when WAT was treated with AZT + 3TC + DTG. Importantly, disruptions in WAT remodeling due to impaired proliferation or adipogenesis may contribute to an increased risk of insulin resistance and other metabolic complications.^(37)^

Leptin is a hormone primarily secreted by adipose tissue and reflects the total body fat mass. It acts predominantly on specific areas of the hypothalamus, where it suppresses food intake and promotes energy expenditure. Obese individuals typically present with elevated leptin levels, which enhance the secretion of pro-inflammatory cytokines and contribute to their state of low-grade inflammation and higher risk of developing other diseases, such as type 2 diabetes.^(38)^ As expected, patients with lipoatrophy induced by earlier antiretroviral therapy regimens exhibited reduced expression of leptin in adipose tissue.^(39)^ Interestingly, *in vitro* studies have shown that treatment of SVF cells from subcutaneous WAT or SGBS preadipocytes induced to differentiate into adipocytes with DTG alone led to decreased leptin secretion,^(30,32,33)^ which was not reversed by co-treatment with TDF.^(30)^ In contrast, our results demonstrate that *in vitro* treatment of subcutaneous WAT with TDF + 3TC + DTG increased leptin expression. This finding aligns with a study in macaques infected with simian immunode-ciency virus (SIV) and treated with TDF + FTC + DTG that reported higher leptin expression in subcutaneous, but not visceral WAT than in untreated SIV-infected animals.^(40)^ These observations suggest that the effects of antiretrovirals on leptin regulation may differ depending on whether the treatment is applied to isolated cells or to the entire tissue or organism. Although it remains unclear whether individuals on current antiretroviral regimens have indeed higher leptin plasma levels, this could suggest an increased risk of developing metabolic dysfunction, as observed in obesity.

Adiponectin is a hormone secreted by adipocytes that plays a critical metabolic role, enhancing insulin sensitivity and exerting anti-inflammatory and anti-atherosclerotic effects.^(41)^ In individuals living with HIV, earlier antiretroviral therapy regimens associated with lipodystrophy have been linked to reduced adiponectin expression in subcutaneous WAT compared to that in HIV-seronegative controls^(39,42)^ or individuals on antiretroviral therapy regimens without lipodystrophy,^(43,44)^ with reduced adiponectin correlating with insulin resistance. Moreover, DTG treatment has also been associated with lower plasma adiponectin concentration in people living with HIV with high cardiovascular risk who switched from PI to DTG-based regimens.^(45)^ Supporting this, adiponectin expression and secretion were reduced in SVF cells from subcutaneous WAT and in SGBS preadipocytes induced to differentiate into adipocytes and treated with DTG alone compared to that in the control,^(30,32,33)^ which was associated with insulin resistance^(32)^ and not reversed by co-treatment with TDF.^(30)^ However, herein, no alterations were observed in adiponectin secretion measured by ELISA or gene expression assessed by qPCR. This discrepancy may be explained by the combination of DTG with TDF and 3TC or by the influence of other cells present in adipose tissue on adiponectin expression by adipocytes.

Adipose tissue also plays a crucial role in regulating systemic inflammation through the secretion of pro- and anti-inflammatory cytokines. Pro-inflammatory cytokines, such as IL-6 and TNFα, secreted by adipocytes and immune cells within adipose tissue, contribute to insulin resistance and metabolic dysfunction,^(46)^ with elevated levels observed in conditions, such as type 2 diabetes and obesity.^(47)^ Conversely, IL-10 is an anti-inflammatory cytokine produced by adipose tissue and linked to increased insulin sensitivity.^(48)^ Earlier antiretroviral regimens have been associated with increased expression of pro-inflammatory cytokines, such as TNFα and IL-6, in subcutaneous WAT, particularly in individuals with lipodystrophy.^(39,49–51)^ It has been reported that in visceral WAT from people living with HIV on earlier antiretroviral therapy regimens, TNFα expression is increased, whereas IL-6 expression is downregulated compared to that in healthy controls.^(43)^ Additionally, DTG treatment reduced IL-6 expression in SGBS adipocytes, while TDF had no effect.^(30)^ In contrast to these previous reports, our study found no alterations in inflammatory cytokine expression, suggesting a minimal impact of the tested antiretroviral combination on adipose tissue inflammatory responses. Additionally, the unchanged cytokine profile observed herein may reflect a dynamic interplay between pro- and anti-inflammatory signals. For instance, a potential increase in IL-10 could suppress the expression of TNFα and IL-6, which in turn might lead to a feedback inhibition of IL-10 itself. Further studies using additional time points and complementary methodologies are needed to better elucidate these regulatory mechanisms.

Lipolysis involves the breakdown of triglycerides stored in lipid droplets within adipocytes and results in the release of glycerol and free fatty acids, which serve as energy source.^(15)^ Earlier antiretroviral regimens have been associated with increased lipolysis in HIV-positive individuals, particularly in those with lipodystrophy, compared to that in HIV-negative individuals.^(52,53)^ *In vitro* treatment of 3T3-L1 adipocytes with AZT alone or in combination with 3TC, but not with TDF or 3TC alone, reduced lipid accumulation, potentially through higher rates of lipolysis.^(29)^ Supporting this, SGBS adipocytes treated with TDF also exhibited no alteration in lipolysis.^(30)^ In contrast, DTG reportedly reduces glycerol release from SGBS adipocytes^(30)^ as well as from human and mouse SVF cells induced to differentiate into beige or brown adipocyte,^(34,35)^ suggesting an inhibitory effect of DTG on lipolysis. Herein, however, no alteration in lipolysis was observed, indicating that when different antiretrovirals are combined, their individual effects on lipolysis may be mitigated.

This study has limitations. There was no analysis of the effects of each antiretroviral drug individually, as we evaluated only their combined use. Future investigations should aim to dissect the specific contributions of each drug to the observed biological effects, which could support the development of therapeutic regimens that preserve efficacy while minimizing adverse outcomes. Moreover, antibiotics were used in the culture medium. While essential to prevent contamination, antibiotics are known to influence cellular behavior, including proliferation, differentiation, and gene expression, and may have interfered with the cellular responses to antiretroviral therapy in our experiments. Furthermore, we did not perform phenotypic characterization of the SVF cells affected by antiretroviral therapy. As a result, we cannot determine whether the observed effects were general or restricted to specific cellular subpopulations. Future studies employing flow cytometry or immunostaining techniques are warranted to address this gap.

## CONCLUSION

Our findings indicate that both antiretroviral combinations tested, *i.e*., TDF + 3TC + DTG or AZT + 3TC + DTG, reduced the number of SVF cells from subcutaneous and visceral adipose tissue. Moreover, TDF + 3TC + DTG increased FABP4 and leptin expression in subcutaneous adipose tissue. No significant alterations were observed directly in inflammatory cytokine expression, adiponectin expression and secretion, or lipolysis. These results provide valuable contribution to the direct effects of contemporary antiretroviral regimens on adipose tissue, raising questions about their metabolic safety in people living with HIV. Therefore, further studies are necessary to explore the long-term implications of these treatments *in vivo*.

## Data Availability

Data are available to reviewers upon request.
